# 2D hexagonal boron nitride (h-BN) nanosheets in protective coatings: A literature review

**DOI:** 10.1016/j.heliyon.2023.e19362

**Published:** 2023-08-25

**Authors:** Viswanathan S. Saji

**Affiliations:** Interdisciplinary Research Center for Advanced Materials, King Fahd University of Petroleum & Minerals, Dhahran - 31261, Saudi Arabia

**Keywords:** Boron nitride nanosheets, Chemical vapour deposition, Polymer nanocomposites, Protective coatings, Anti-oxidation and anti-corrosion

## Abstract

The layered 2D hexagonal boron nitride (h-BN) nanosheets (BNNSs) have received significant attention as effective fillers for composite protective coatings in anti-corrosion, anti-oxidation and anti-wear applications. Vapour deposited h-BN mono/multilayers are related classes well-recognized as protective thin films and coatings. This review comprehensively accounts for the research and development of BNNSs in protective coatings. Chemical vapour deposited (CVD) BN thin films and exfoliated BNNSs-incorporated composite polymer coatings are primarily discussed. Inorganic and nanocarbon-based composite coatings are also covered. Future research potentials are presented.

## Highlights

• Research updates on vapour-deposited h-BN for anti-corrosion, anti-oxidation and anti-wear coatings are presented.
• Applications of h-BN nanosheets incorporated inorganic, nanocarbon and polymer-based coatings are detailed.
• Exfoliated h-BN nanosheets incorporated epoxy coatings are highlighted.

## 1 Introduction

**Fig. 1:**
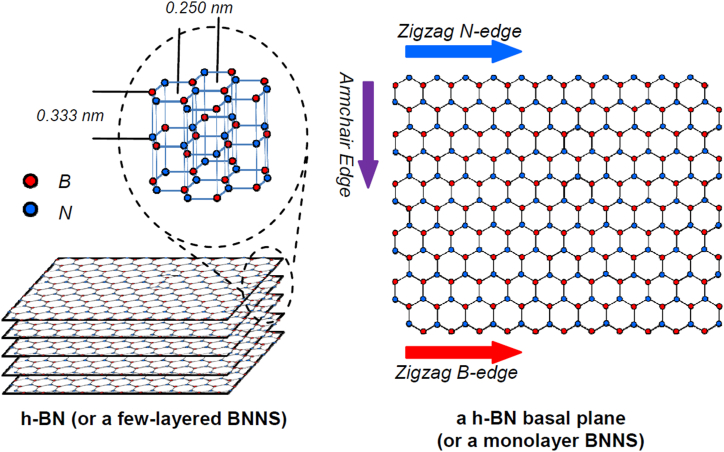
2D h-BN nanosheet structure: (Left) Few-layered and (Right) Monolayer. *Reproduced with permission from**Ref. [8]**© 2012, The Royal Society of Chemistry*.

The layered 2D nanosheets (NSs) with thickness <10 nm and lateral size >100 nm are well recognized as exceptional nanomaterials due to their greater specific surface area and striking chemical, thermal and mechanical properties for potential applications in energy storage materials, catalysis, electronics, water purification and protective coatings. Since graphene (GR)'s discovery, a wide range of 2D nanomaterials has been explored, including layered hexagonal BN (h-BN), graphitic carbon nitride, transition metal dichalcogenides, MXenes, layered double hydroxides and 2D allotropes of Si, Ge, P etc. [[1], [2], [3], [4]]. Being analogous to GR and electrically insulating, the h-BN NSs (BNNSs), also known as white GR, have attracted significant recent research attention, particularly in protective coatings. The h-BN is the most predominant form of crystalline BN, the other being cubic and wurtzite. Akin to carbon nanotubes (CNTs) and GR, the h-BN exists in 1D BN nanotubes and 2D BNNSs [5,6]. h-BN has a 2D honeycomb lattice alternately arranged by N and B atoms, with B–N covalent bonds of 1.45 A^°^ and BN interlayer spacings of 0.333 nm detained by weak van der Waals forces (Fig. 1) [2,7,8]. The bonding in h-BN is analogous to aromatic compounds; however, the high electronegativity of nitrogen and the associated partial ionic properties make it an electrical insulator with a large band gap [[1], [2], [3]].

BNNSs can be realized via bottom-up or top-down approaches. The typical top-down methods are exfoliation processes, either liquid phase (solvent intercalation, hydrothermal, thermal shocking, microwave-assisted, supercritical etc.) or mechanical (scotch tape, ball milling etc.). The major bottom-up processes are vapour deposition (PVD and CVD), laser deposition, surface segregation and molecular beam epitaxy. The exfoliation techniques are advantageous in fabricating high-quality crystalline NSs; however, the percentage yield might be low; also, the probability of layer stacking exists, whereas bottom-up approaches, such as PVD and CVD, are precisely controllable processes [[9], [10], [11], [12]]. The surface area of few-layer BNNSs is significantly higher (∼2600 m^2^/g) compared to that of BN nanotubes (212–254 m^2^/g) and bulk BN (∼10 m^2^/g). They have superior thermal conductivity (∼2000 W/m·K) and thermal stability (>800 °C in the air) [13,14]. BNNS is advantageous to several 2D counterparts due to its better thermal and chemical stability, good light transmittance, and the insulating characteristics. The high thermal and chemical stability is attributed to the strength of σ bonds, partial ionicity, and lack of surface states and dangling bonds [12,15]. BNNSs have exciting potential applications in insulating coatings, corrosion/oxidation/wear-resistant coatings, thermal conductive composites, lubricants, water treatment, hydrogen storage, packaging industries, electrical and electronic applications, etc.

The higher oxidation resistance (>800 °C) over GR (250 °C) makes BNNSs an attractive candidate for high-temperature oxidation resistance coatings [16]. The electrical insulating property and the absence of potential galvanic corrosion are attractive attributes compared to the conducting GR. The layered structure, significant gas/liquid impermeability, and brilliant barrier property are added advantages [17]. A comparative study on the oxidation of Cu coated by either GR or h-BN single-layers has shown that both GR and h-BN coatings provided good short-term (30 min, <250 °C) oxidation protection, with the GR coatings surpassing the h-BN, which was attributed to the more prominent grains and fewer wrinkles with GR. However, GR failed after a few hours, where the electrically insulating h-BN eventually outperformed GR; the long-term protection was credited to the absence of galvanic corrosion with the h-BN coating [18]. The electrical insulating characteristics and superior mechanical and barrier properties make BNNSs excellent fillers for composite coatings. One major drawback is their tendency to agglomerate, demanding a good dispersion strategy for coating applications. BNNS's properties could be well-tuned via doping, substitution, functionalization and hybridization approaches [[9], [10], [11],^19^,^20^].

**Fig. 2:**
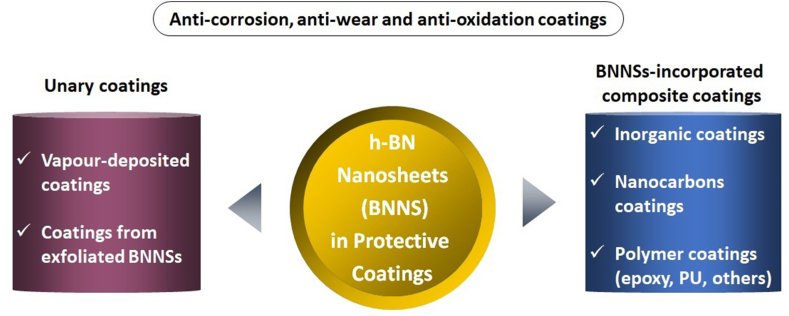
A scheme showing of the review focus.

A few reviews on 2D nanomaterials-based anti-corrosion coatings, particularly polymer composite coatings, have addressed BNNSs as a part [3,[21], [22], [23]]. Yang et al. summarized the fabrication techniques of h-BN and GR and their potential and challenges for protective coatings [16]. A dedicated review focusing on BNNSs in protective coatings needs to be included in the current literature. Here, we provide the most recent advances in 2D h-BN-based anti-oxidation, anti-wear and anti-corrosion coatings. Fig. 2 shows a schematic of the review focus. Section 2 focuses on CVD-made 2D h-BN films. Section 3 covers BNNSs-incorporated composite coatings.

## 2 BNNSs coatings

### 2.1 Vapour deposited coatings

Vapour deposition methods, particularly the CVD, are the best approach to fabricating high-quality 2D single and multilayer BN thin films and coatings. In CVD, gaseous reactants chemically react on or nearby the surface of a heated substrate, forming mono/multilayer or functionally graded thin films with large-area uniformity and first-rate layer-number selectivity [ [11]].

Paffett et al., in 1990 reported for the first time the adsorption and decomposition of borazine (B_3_N_3_H_6_) on Ru(0011) and Pt(111) [24]. Nagashima et al., in 1995 deposited h-BN films on Ni(111), Pt(111) and Pd(111) via decomposition of borazine at 700–800 °C [7,25]. Shi et al. made h-BN films of 5–50 nm thickness and 20 μm lateral size on polycrystalline Ni [26], and Song et al. made h-BN films of 2–5 atomic layers on Cu [27]. Liu et al. reported large area crystalline h-BN atomic layers (2–5 nm, CVD at 1100 °C, 50 min, Ar/H_2_ and NH_3_–BH_3_) on Ni foil as superior oxidation-resistant coatings [28]. Kidambi et al. have shown that the h-BN layer nucleation and growth on polycrystalline Cu happen isothermally during borazine exposure. The study showed that B is integrated into the bulk Cu, while N is not [29]. Strong temperature-dependence of the h-BN crystal size was studied by Stehle et al., larger-sized flakes formed at higher temperatures [30]. Ren et al. in their studies on grain boundaries in monolayer polycrystalline h-BN, revealed that most of the grain boundaries were formed via adjacent grain's overlapping, different from the covalently constructed grain boundaries characteristic of 2D materials. DFT studies showed that hydrogen is indispensable in overlapping grain boundary creation [31]. h-BN monolayer has impenetrability to gaseous molecules; the chemically adsorbed atomic oxygens can block the vacancy sites of the monolayer and can play an essential role in the repair [32].

**Fig. 3:**
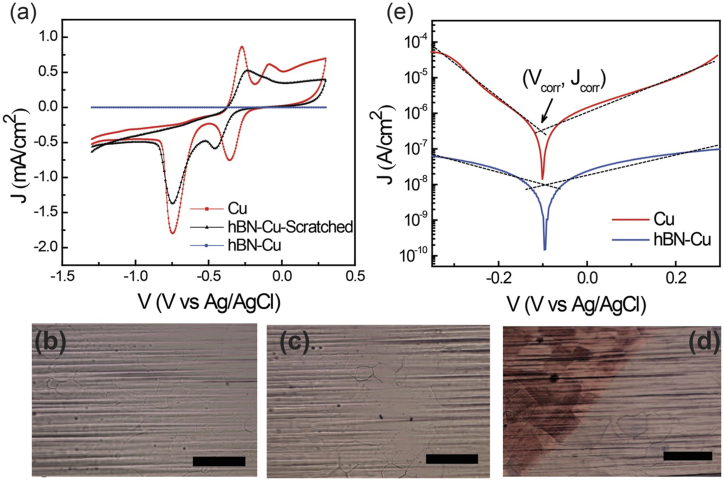
(**a**) CVs of BN-Cu (blue), scratched BN-Cu (black) and plain Cu (red) in 0.1 M NaOH. Optical images of BN-Cu before (**b**) and after (**c**) 30 successive CV scans. (**d**) Corresponding image of scratched BN-Cu after 30 CV scans (scale bar - 100 μm). (**e**) Tafel plots. *Reproduced from**Ref. [35]**under creative commons CC-BY © 2017 The Author(s)*.

CVD (NH_3_–BH_3_, 1050 °C, 10 min) grown large-area h-BN thin film on Ni foil was transferred onto sputtered Cu via a polymer-assisted wet transfer and shown to be an excellent corrosion passivation coating [33]. A long-term (160 days) study in an ambient environment showed that the monolayer h-BN has much improved barrier effect than GR, attributed to the insulating characteristics and more excellent impermeability, which overwhelm the galvanic corrosion [34]. The protection capability of monolayer h-BN (NH_3_–BH_3_, 1000 °C, thickness - 0.45 nm) for Cu in 0.1 M NaOH is evident from Fig. 3. The two anodic and cathodic peaks (corresponding to redox reactions of Cu) observed with the bare Cu were absent with the monolayer covered Cu, even after 30 successive cyclic voltammetry (CV) scans. Compared to the plain Cu, the monolayer covered Cu's cathodic and anodic currents were reduced by more than three orders of magnitude (Fig. 3a). Upon mechanically scratching the film, the redox peaks reappeared as expected. Before and after 30 CV sweeps, optical images presented no significant changes with the monolayer covered Cu (Fig. 3b and c). The corresponding image of the scratched surface was distinctly different (Fig. 3d). The corrosion current density (*i*_corr_) of the monolayer covered sample was nearly one order of magnitude inferior to plain Cu (Fig. 3e) [35]. Electrochemical and DFT studies on h-BN coated Cu in air, oxygen, water and salt solution have shown that water, not oxygen, is liable mainly for Cu oxidation. Once entered in the film/Cu interface, water can easily dissociate with an energy release of 1 eV. In 0.5 M NaCl, compact and thin films (1–2 atomic layers) with larger domains and fewer defects provided excellent protection for Cu, while a thick multilayered film with cracks and grain boundaries failed. Many pits formed on the plain Cu, whereas the morphology of the h-BN covered Cu appeared mostly unaltered after the corrosion tests [36].

Several works compared the oxidation and corrosion resistance of CVD-made GR and h-BN films. The higher proportions of surface atoms in thin films make them prone to oxidation; hence, the thermal stability in air strongly depends on film thickness. It has been shown that monolayer GR reacted with oxygen gas at 250 °C and subsequently got etched at 450 °C, whereas bilayer and few-layer GR were not etched till 500 °C. In contrast, an h-BN monolayer showed no etching up to 840 °C. Long-term oxidation studies (250 °C, air, 20–100 h) on monolayer and 20-layer BNNSs covered Cu foils showed that after heating, the plain Cu (after 20 h) and monolayer roofed Cu (100 h) turn into virtually black, with larger oxide particles, while the 20-layer BNNSs covered Cu became marginally darker only, with minor oxide particles. Their elemental analysis confirmed that the 20-layer h-BN diminished ∼90% of the Cu oxidation, whereas the monolayer had no protection. Electrochemical studies in 0.1 M NaCl suggested a higher corrosion resistance for the thicker NSs covered Cu [14]. Ren et al. on alternating temperature tests (30 days of air exposure with subsequent heating at 200 °C), showed that the oxidation advanced gradually in multilayer as the underlying defects were almost completely covered, suppressing the vertical oxygen diffusion; oxidation mainly occurred on the wrinkle's region. On the other hand, with the monolayer covered Cu, the oxidation happened primarily in the h-BN grain boundaries and the layer structural defects. Electrochemical studies revealed that the h-BN layer acted as an effective physical barrier in 3.5% NaCl and prevented electron diffusion owed to its electrical insulativity [37]. XPS studies on the comparative protective action of CVD-made GR and h-BN towards Cu oxidation have shown that the protection kinetics is dissimilar at the two interfaces. The hot Cu surface is screened from oxygen very well by the insulating h-BN, even though certain oxygen intercalates via boundary regions or wrinkles until up to 300 °C. With GR/Cu, instead, oxygen starts to intercalate underneath the GR sheets, and sluggish Cu oxidation starts at ∼220 °C, attributed to the galvanic action [38]. Stability studies of mono and multilayer h-BN in aqueous H_2_O_2_ (11 h) at room temperature demonstrated that the monolayer h-BN is as ineffective as GR. The monolayer h-BN was also ineffective in the air atmosphere for longer durations (230 days). AES and SIMS studies recommended that the oxygen diffusion to the metallic substrate could happen through h-BN's grain boundaries; whereas a saturation could be achieved as the oxidized area was not increased after a limit, making h-BN multilayer films apt for longstanding oxidation protection; as it could provide more effective diffusion barrier. A multilayer h-BN film with 5–7 layers showed good protection [39].

These studies proved that CVD-made few-layer h-BN films are excellent anti-corrosion and anti-oxidation coatings, especially for metals like Cu, suitable for long-term applications. However, monolayer scale films were not found to be effective where oxidation initiated quickly along the grain boundaries. The insufficient surface coverage and the defective sites contributed to inferior long-term performance. Precise layer count and film thickness optimization are essential for better protection. On the other hand, monolayer films are efficient in anti-bacterial applications, as discussed below.

**Fig. 4:**
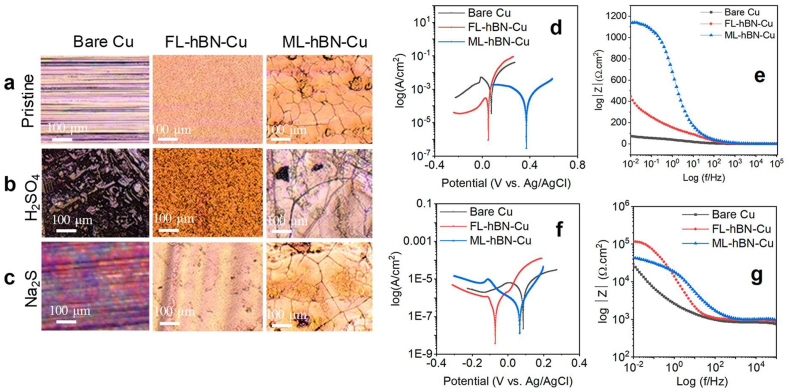
Optical images of **(a)** Cu, FL-BN-Cu and ML-BN-Cu **(b, c)** after exposed to **(b)** H_2_SO_4_ and **(c)** Na_2_S media. Tafel and Bode magnitude plots in **(d, e)** 0.5 M H_2_SO_4_ and **(f, g)** 0.1 M Na_2_S. *Reproduced with permission from**Ref. [42]**© 2020 American Chemical Society*.

Monolayer h-BN is shown to be effective for anti-bacterial and anti-biocorrosion applications. Comparative studies on bacterial interactions with Cu substrates coated with monolayer h-BN and GR have demonstrated that the latter has the same effect as the as-grown GR and effectively blocks bacterial activity and Cu biocorrosion. Their result suggests no correlation between the conductivity of the layer and their anti-bacterial activity [40]. Single-layer h-BN was reported as the thinnest insulating fence on Cu to microbial corrosion by *D*. *alaskensis (SRB)*. CV results showed that single-layer h-BN served as an impervious barricade to destructive metabolites even at high potentials of 0.2 V (*vs* Ag/AgCl), reducing the peak anodic current by ∼36 times. The single-layer film offered ∼91% protection efficiency, analogous to a thicker polyaniline coating. The rate of biocorrosion in the single-layer h-BN sample was ∼67% lesser on the first day and ∼87% lesser after 24 days. A comparative study with GR showed that the galvanic current density for single-layer GR/Cu was ∼392 times greater than single-layer h-BN/Cu (∼8.4 × 10^−2^ μA/cm^2^). Their H_2_O_2_ oxidation studies disclosed that the bare Cu and single-layer h-Bn covered Cu were oxidized by ∼71% and 45%, respectively. Raman mapping results indicated a surface coverage area of ∼88% for the single-layer [41]. The authors in a subsequent work showed that CVD-made (liquid B_3_N_3_H_6_, 750 °C, 30 min) few-layered (∼4 layers, FL) and multilayered (∼9 layers, ML) h-BN provided ∼6–7 times reduced corrosion rate compared to bare Cu both in abiotic (H_2_SO_4_ and Na_2_S) (see Fig. **4**) and biotic (*D. alaskensis*) media. As the optical images revealed, the coated surface sustained undamaged compared to bare Cu (Fig. 4a-c). In H_2_SO_4_, the corrosion rates of ML-BN-Cu (33.72 mpy) and FL-BN-Cu (35.25 mpy) were ∼14 times lesser to plain Cu (484 mpy) (Fig. **4d**). As evident from the impedance spectroscopy (EIS) Bode magnitude plots, both ML-BN-Cu and FL-BN-Cu reduced the corrosion rate by ∼2 orders of magnitude than bare Cu (Fig. **4e**). In sulfide medium, the corrosion rates of ML-BN-Cu (0.29 mpy) and FL-BN-Cu (0.21 mpy) were ∼12 and 17 times lesser to the bare (3.53 mpy) (Fig. 4f). The low-frequency impedance values of ML-BN-Cu and FL-BN-Cu were 2 and 8 folds higher (Fig. 4g). Their studies in the biotic environment revealed a similar result, where the samples efficiently protected the Cu surface from biocorrosion during 48 h of *D. alaskensis* exposure and 650 h of its sessile counterpart exposure. The biocorrosion rates of FL-BN-Cu and ML-BN-Cu were an order of magnitude inferior to bare Cu. The biocorrosion rates of ML-BN-Cu augmented from 0.04 mpy (day 0) to 1.57 mpy (day 27), whereas the biocorrosion rate of FL-BN-Cu increased marginally and stabilized at 0.31 mpy (day 27). DFT studies demonstrated enhanced adsorption energy of sulfur compounds at point defects such as edges, vacancies, substitutional atoms and grain boundaries, which can worsen the protection [42].

EIS analysis of CVD (B powder and NH_3_, 1200 °C) BNNSs coated (5–6 nm) mild steel displayed charge transfer resistance (*R*_ct_) of 1996.3 Ω cm^2^ compared to 298.8 of bare steel [19]. A coating made using commercially available CVD-grown BNNSs on Cu reduced the *i*_corr_ to 0.27 μA/cm^2^, compared to bare Cu's 0.93 μA/cm^2^ [43].

Several earlier works on PVD h-BN films are available, including radio-frequency magnetron sputtered BN coating for Al corrosion [44], BN film by ion beam and vapour deposition [45], BN coating as hydrogen permeation barriers for stainless steels [46], and vacuum plasma spray coated composite coatings of TiAl/BN and Ti_3_Al/BN [47], and Al-BN [48]. Tang et al. in a recent work, demonstrated that the sputtering power and negative bias voltage during magnetron sputtering have an important role in the densification of h-BN thin film (∼200 nm on 304 SS). The nanolayer resisted steel's oxidation in the air up to 600 °C for 30 min. Durable passive corrosion protection was noted during 10 weeks of immersion in 3.5 wt% NaCl. The *i*_corr_ determined by Tafel analysis for bare and coated steel were 2.29 and 0.74 μA/cm^2^, respectively. The coating significantly enhanced the hydrophobicity, from 41^°^ on the bare surface to 119^°^ for the coated sample. The durability over six months of salt water immersion demonstrated the potential for long-term use [49].

All these reports emphasize the upper hand of h-BN over GR. CVD is a powerful tool for generating high-quality h-BN thin films suitable for anti-oxidation and anti-corrosion applications. The below section discusses a few works where exfoliated BNNSs were used for coating fabrication.

### 2.2 Coatings from exfoliated BNNSs

**Fig. 5:**
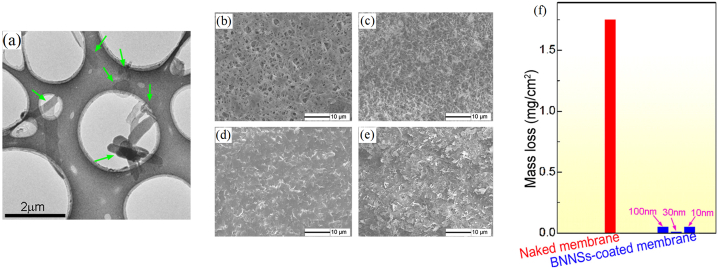
**(a)** TEM image of BNNSs. **(b**–**e)** SEM surface images **(b, d)** before and **(c, e)** after corrosion studies. The images **(b)** & **(c)** correspond to uncoated polymer and **(d)** & **(e)** correspond to the BNNSs coated polymer. **(f)** Mass loss of pristine and coated (with different-sized BNNSs) polymer membranes. *Reproduced with permission from**Ref. [50]**© 2014 AIP Publishing LLC.*

Oxygen atoms can degrade polymeric components. BNNSs coatings were explored for safeguarding polymers from oxygen-atom corrosion. Here, liquid-exfoliated BNNSs were assembled into a coating by vacuum filtration. The exfoliated BNNSs were several hundred nanometers large, with a 0.5–4.2 nm thickness (Fig. 5a). The virgin polymer membrane displays a porous surface morphology with firmly stacked solid nylon fibres (Fig. 5b). In the coated membrane, the NSs are closely allied in the plane resulting in a uniform coating (Fig. 5d). The virgin polymer undergoes severe corrosion with substantial mass loss during oxygen-atom exposure in a filament-discharge facility, whereas the BNNSs coated polymer sustained undamaged (Fig. 5c and e), even with a 10 nm coating. The mass loss variation after exposure in a total oxygen-atom flux of ∼2.78 × 10^20^ atoms/cm^2^ is provided in Fig. 5f. The two effects that contributed to the anticorrosion mechanism of the coated substarte were suggested to be the barrier effect and the bonding effect (large surface areas BNNSs are prone to form bonds with oxygen atoms) [50].

Teng et al. fabricated a robust hydrophobic BNNSs coating via liquid exfoliation and interface self-assembly. The coating can prevent Cu substrate from oxidising at temperatures as high as 500 °C. A dispersion of exfoliated BNNSs was inoculated into the water/air interface at an optimum speed, and the NSs self-assembled into a film owed to the Marangoni effect, which was transferred onto the Cu surface. After a fluorosilane modification, the hydrophobic film could effectually guard the substrate against high-temperature oxidation [51].

## 3 BNNSs incorporated composite coatings

The primary application of BNNSs in protective coatings is as fillers in composite coatings. The nanofiller effect is well known as it can enhance a composite coating's cohesion, uniformity and thickness. Nanofillers can inhibit components from being pulled out, homogenize the stress and improve the load-carrying capacity. The high aspect ratio of the exfoliated and functionalized BNNSs could facilitate greater surface area and better interfacial molecular interaction in the composite matrix. On the other hand, the BNNSs could act as a matrix and serve as a heterogeneous nucleation and growth site for nanocrystals [1,13,[52], [53], [54]]. This section discusses reported works on BNNSs-incorporated inorganic, nanocarbon and polymer-based nanocomposite coatings.

### 3.1 Inorganic coatings

#### 3.1.1 Chemical conversion coatings

Chemical Conversion coatings are primarily employed as the first layer of industrial multilayer coatings [[55], [56], [57]]. BNNSs could be incorporated into the conversion coatings to enhance the protective effect. It has been shown that BNNSs significantly improved the corrosion protection of a zinc phosphate coating for mild steel. BNNSs in the coating bath (0.45 g/L) helped decrease the phosphate crystal size, making the composite layer denser and finer. The deposition yield increased from 15.83 g/m^2^ (without BNNSs) to 25.10 g/m^2^ at 0.45 g/L of BNNSs concentration. The *i*_corr_ of the BNNSs-zinc phosphate coated sample (3.21 × 10^−6^ A/cm^2^) in 3.5 wt% NaCl was an order of magnitude lesser than the sample coated without BNNSs. The excellent surface adsoption of BNNSs was attributed to the greater specific surface area and the presence of hydroxyl groups. The BNNS's zeta potential in water (−61.33 mV) benefited surface adsorption through electrostatic interactions [58].

Suitable functionalization can overcome the chemical inertness of BNNSs. Several works utilized poly(dopamine) (PDA) for the surface modification of BNNSs (see Section 3.3). Muhammad et al. used PDA to improve the dispersibility of BNNSs and modify the chemical interaction between zinc phosphate conversion coating and a silane coupling agent (KH560). The NSs were stirred in a solution of tris(hydroxyethyl aminomethane), ethanol and dopamine hydrochloride at 60 °C to improve the dispersibility, slow down the PDA polymerization rate and prevent NSs aggregation. The PDA-modified BNNSs (0.60 g/L) enhanced the mass per unit area of the coating up to 26.8 g/m^2^, compared to 15.2 g/m^2^ of the coating without BNNSs. The NSs addition in the bath also helped enhance the hopeite growth rate. Compared with the bare sample, the *i*_corr_ of the composite-coated mild steel sample in 3.5 wt% NaCl was reduced by one order of magnitude (1.95× 10^−5^ A/cm^2^) [59].

**Fig. 6:**
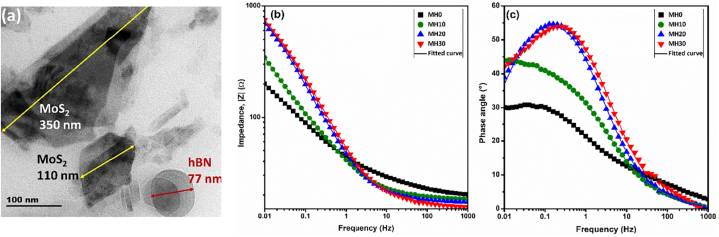
(a) HRTEM images of BNNSs-MoS_2_ hybrid (MH30). **(b, c)** Impedance spectra of the MoS_2_ (MH0) coating with different BNNSs (0–30 wt%) content. *Reproduced with permission from**Ref. [61]**© 2022 Elsevier B.V.*

MoS_2_ is well known as a solid lubricant owing to its superior wear resistance characteristics. However, they are prone to oxidation and corrosion when exposed to oxygen and humid air. A few reports explored MoS_2_-BNNSs coatings. The corrosion resistance enhancement was mainly credited to the h-BN's insulating property [60,61]. Joseph et al. examined the effect of BNNSs varying concentrations on the corrosion and antifriction performance of MoS_2_-BNNSs hybrid coatings for mild steel. MoS_2_ and BNNSs were prepared from the bulk via liquid phase exfoliation. The HRTEM image of the mixed NSs powder evidence a few layers of MoS_2_ (100–500 nm) and h-BN flakes (60–120 nm) (Fig. 6a). The more miniature h-BN sheets can inhabit the potholes, pores and spaces amid the MoS_2_ flakes, thus lowering the porosity and surface roughness while augmenting the coating density. Excellent coating adhesion was observed in cross-hatch analysis. The increase in h-BN content in the composite boosted the corrosion protection, as evident from the EIS Bode plots (Fig. 6b and c). Raman spectroscopy demonstrated strong interlayer coupling between h-BN and MoS_2_ layers, reducing the charge transfer between the layers and impeding corrosion and oxidation [61].

#### 3.1.2 Electro/electrophoretic deposition

**Fig. 7:**
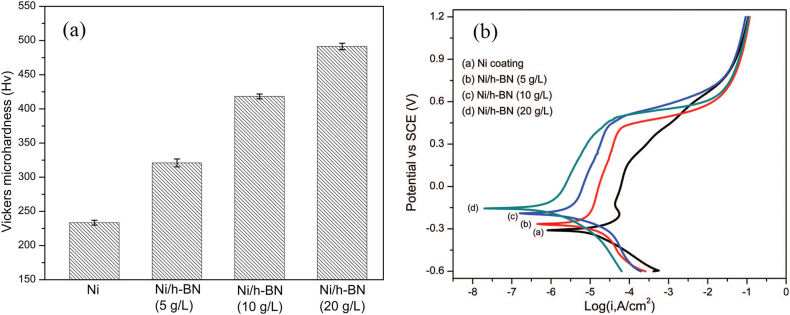
(a) Vickers microhardness and **(b)** Potentiodynamic polarization curves of Ni and Ni-BNNSs coatings. *Reproduced with permission from**Ref. [64]**© 2012 The Electrochemical Society.*

Electrodeposition (ED) [62] and electrophoretic deposition (EPD) [63] of composite coatings encompassing fine ceramics second-phase particles into metal matrix have fascinated widespread attention due to the enhanced mechanical, anti-corrosion and anti-tribological properties. Ni-matrix composite coatings were well fabricated by incorporating BNNSs (5–20 g/L) using a sulfamate bath via pulse ED (80 mA/cm^2^, 100 Hz, 50% duty cycle). The composite coating presented a finer-grained smooth surface compared to pure Ni coating. A maximum microhardness of ∼490 Hv was reached when the deposition bath contained 20 g/L BNNSs (Fig. 7a), attributed to the refining nickel crystallites, dispersive strengthening, and hindering of grain boundary sliding and dislocation motion by the BNNSs. Electrochemical studies evidence significantly improved corrosion resistance in 3.5 wt% NaCl, as seen in Fig. 7b. The composite coating demonstrated a broader passivation region and positive shift of corrosion potential compared to plain Ni, with significantly reduced *i*_corr_ [64].

Chitosan (CS) - BNNSs coating was deposited by EPD on a Mg alloy to reduce alloy degradation and improve antibacterial activity for bone implant applications. The alloy sample was first subjected to plasma electrolytic oxidation (200 V, 800 Hz, 15% duty cycle) to fabricate a thicker oxide layer coating. A stable suspension of 0.5 g/L CS dissolved in isopropanol (30 vol%) and acetic acid (0.5 vol%), with added 1 g/L BNNSs was used for the EPD (0.14 mA/cm^2^, 240 s). The weight loss after 7 days of soaking in SBF was ∼5 mg/cm^2^ compared to 25 mg/cm^2^ of the bare sample. Tafel analysis showed that the composite-coated sample exhibited the lowermost corrosion rate, *i*_corr_ decreased from 6.65 × 10^−4^ A/cm^2^ to 7.56 × 10^−6^ A/cm^2^. BNNSs addition helped avoid forming large rupture areas and enhanced the hydrophobicity. The composite coating had a better antibacterial performance (decreased viability of *E. coli* and *S. aureus*) due to the h-BN nanospike's bactericidal effect. Cytocompatibility studies revealed that the composite layer had no negating effect on mouse osteoblast adhesion and proliferation [65]. Zhao et al. used gas-assisted liquid exfoliated and phosphorylated BNNSs for EPD on Q235 carbon steel. The *R*_ct_ values of the bare sample and the samples coated at 1.2, 1.0, 0.8 and 0.6 V (*vs* SCE) were 3.72 × 10^3^, 1.01 × 10^5^, 1.38 × 10^5^, 9.76 × 10^4^ and 8.54 × 10^4^ Ω cm^2^, respectively. The better corrosion resistance was credited to the more significant barrier effect of BNNSs and phosphate-assisted passivation [66].

#### 3.1.3 Sol-gel coatings

Zirconium oxide coating doped with BNNSs has been sol-gel deposited on 316L SS and heat treated at 120 °C. The hydrophilic zirconium oxide coating (water contact angle ∼ 65^°^) becomes hydrophobic after BNNSs incorporation (102^°^). The barrier protection of the composite coating varied with the size and distribution of BNNSs. When the NSs average size was less than the optimum size required for the best barrier property, the pores in the composite coating were not sealed. At a suitable filler size, the BNNSs fill the coating's pores, diminish the oxidation kinetics, and restrict the penetration pathway. At a further larger filler size, the NSs efficiently enhanced the barrier effect, where the aggressive species has to take zig-zag ways to approach the coating/substrate interface, improving the corrosion resistance considerably [67]. The result supports the need for optimising the NS's aspect ratio for better barrier property.

### 3.2 Nanocarbons coatings

Due to the analogous atomic size and structure, carbon doping (n-doping) of h-BN is a well-known approach to achieving desired electronic and optical properties [68]. Several efforts were made on nanocarbon-BNNS hybrids, mainly in anti-wear coating applications.

BNNS - carboxylated CNT composites with different compositions were prepared by covalent crosslinking using carbodiimide reaction. The fabric composited with the BNNS - CNT displayed noteworthy improvement in thermal stability and tensile strength, superior to pristine fabric and those reinforced by CNT or BNNS. A BN_0.5_C_0.5_ fabric manifested the best anti-wear properties credited to the self-lubricating effect of BNNS and the load-carrying capacity of CNT. 1 wt% of BN_0.5_C_0.5_ incorporation reduced the wear rate and friction coefficient by nearly 64.4% and 32.4%, respectively [69].

Spark plasma sintered GR - BNNS composite provided optimum lubrication characteristics and mechanical properties where BNNS imparted higher hardness, elastic modulus and better wear characteristics, while GR lowered the coefficient of friction [70]. Guimarey et al. spray-coated BNNS - GR (in water-ethanol) on mild steel and demonstrated strong attachments of the NSs to the surface. Compared to the bare sample, the BNNS, GR, and the BNNS - GR treated samples showed wear area decrease of ∼31%, 28% and 44%, respectively. The *i*_corr_ of the samples were in the order: mild steel > GR > BNNS - GR > BNNS. The corresponding polarization resistances were 2092.15, 2157.93, 2438.62 and 3029.95 Ω cm^2^, respectively. The lowest *i*_corr_ and the highest polarization resistance supported the better contribution of h-BN in reducing the corrosion activity of the hybrid. No corrosion was observed on the hybrid-treated sample after six months of air exposure. Significantly enhanced high-temperature oxidation resistance was also noted [71]. A comparative study on 2D graphite, BNNS and MoS_2_ together with PVDF showed that BNNS was far superior to graphite and was marginally inferior to MoS_2_ as an anti-corrosion coating for stainless steel [72].

These reports support that the hybrids of BNNS with CNT, GR or MoS_2_ are suitable candidates for anti-wear and anti-corrosion coatings.

### 3.3 Polymer coatings

Nanocomposite polymer coatings with assimilated BNNSs have a wide range of potential industrial applications. BNNSs can enhance the thermal, mechanical and barrier properties of polymers. Employing proper strategies for homogeneously dispersing the fillers in the coating matrix and improving the cross-linking density could yield superior protective barrier coatings with robust adhesion and reduced defect level [10,13]. The nanocomposite's properties depend on various factors, including aspect ratio and concentration of BNNSs, their dispersion and alignment in the matrix, molecular interactions and stress transfer, interface spacings or voids shaped, and the processing parameters employed [13,54].

The essential steps in fabricating BNNSs - Polymer coatings are (i) exfoliation of h-BN, (ii) covalent/non-covalent functionalization of BNNSs, and (iii) NSs uniform dispersion within the polymer resin. The functionalization step is essential to improve the filler dispersion and filler-polymer interfacial compatibility. The significant criteria for realizing high-performance composite coatings are well-dispersion and parallel assembly of fillers and enhancing the coating's wet adhesion force, cross-linking density, hydrophobicity and self-healing capability [3,73,74]. The different fabrication methods of polymer nanocomposites include (i) melt intercalation/blending (high-temperature annealing of polymer, the addition of nanofillers, and blending), (ii) in-situ polymerization (fillers are mixed with monomers and then polymerized; could allow effective filler dispersion), (iii) solvent mixing (requires a solvent-soluble polymer, fillers are dispersed and intercalated with polymer) and (iv) template synthesis [54]. The BNNS/polymer interphase have a critical role in transferring mechanical stress, thermal heat, and electrical load from one phase into another. Even though the interfacial surface area could be enhanced with a higher loading, the extent of loading above an optimum level typically results in BNNSs aggregation. Basic details of BNNS-polymer interfacial interactions are described elsewhere [3,12,13]. The below section discusses BNNSs-incorporated polymer composite coatings.

#### 3.3.1 Epoxy coatings

**Fig. 8:**
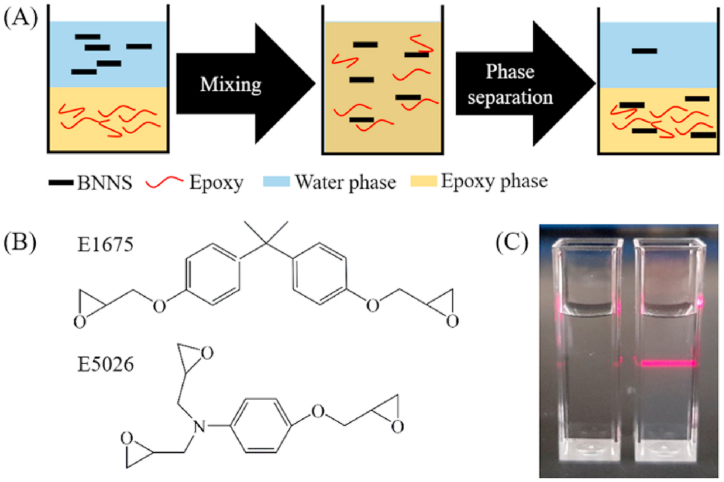
(a) Schematic showing the water - EP transfer of BNNSs. **(b)** Bisphenol-A diglycidyl ether (E1675) and N, N-diglycidyl-4-glycidyloxyaniline (E5026). **(c)** Photograph of (left) water and (right) BNNSs dispersion (0.1 mg/mL aqueous dispersion, laser illuminated, Tyndall effect seen in later). *Reproduced with permission from* Ref. [74] *© 2020 Elsevier Ltd.*

Epoxy (EP) resins are preferred candidates for adhesives and coatings. They are economical and offer decent mechanical properties and chemical, thermal, and weathering durability, which can be tuned via additives and hardeners. The chemical structures of the two widely used EP resins (E1675 and E5026) are provided in Fig. 8b. Fundamental details of EP resins are described elsewhere [75,76].

Incorporating functional nanofillers is a practical way to enhance the properties further. The steps followed in recent work to fabricate (3-aminopropyl)triethoxysilane (APTES) -BNNSs - EP composite coating were (i) ultrasonic exfoliation of h-BN to BNNSs, (ii) functionalization of BNNSs with APTES, (iii) uniform dispersion of APTES - BNNSs in EP by solvent mixing, and (iv) coating of the composite EP coating on the substrate. The composite was prepared by mixing APTES-BNNSs filler and EP in a solvent to achieve uniform dispersion, and then volatilization of the solvent, the addition of a curing agent, vacuum-degassing, and finally, spin-coating and hot-pressing to allow better orientation of the filler in EP matrix. Their fractured surface morphology studies showed that compared to the brittle fracture with smooth EP, the composite coating showed a wrinkled, rough fractured structure, attributed to the local polymer deformation or the matrix shear yielding due to the filler addition. The APTES functionalization helped avoid the filler agglomeration and provided a strong filler/EP interfacial reaction [77]. In addition to the functionalization of the BNNSs, the major parameter determining the BNNSs/EP interaction is the π-π stacking between the hexagonal rings of BNNSs and the benzene rings of EP. Fig. 8demonstrates a scheme used to study EP/BNNSs interactions. The limited compatibility of EP/water leads to phase separation, and the strong EP/BNNSs interaction happens when maximum BNNSs are relocated from water to EP. The study showed that BNNSs interactions varied with the EP resin type; 71.5% of BNNSs were shifted from water to E1675 by mixing, whereas 92.1% of BNNSs shifted with E5026. The results suggested a stronger BNNS - E5026 association, which makes E5026 a potential candidate for the composite formulation. The stronger BNNS/E5026 interaction suggested that the planarity of the EP molecules is critical in determining the extent of filler/EP interactions. In addition, the hydroxyl groups at the BN plane edges and defective sites promoted BNNS/EP interactions through hydrogen bonding. Photographs comparing a dilute BNNSs dispersion to water show that the presence of BNNSs can be confirmed with laser illumination (Fig. 8c) [74].

A few works studied *electrophoretic deposited EP - BNNSs coatings* [78]. Under the electric field, EPD could assist in more ordered dispersion and alignment of BNNSs in the EP matrix. Zhao et al. deposited EP - BNNSs coating on Q235 steel using an emulsion of cationic amino-functionalized BN and cathodic-type waterborne EP at 220 V; the coated sample was dried at 150 °C for 30 min (coating thickness ∼20 μm). SEM images demonstrated ordered and layered microstructure compared to the pristine EP and a casually disseminated coating. After 30 days of salt water immersion, the water uptake of the composite coating was marginal, suggesting that the NSs in the coating matrix improved the barrier properties via tortuous diffusion pathways. The EIS coating resistance of the composite coating (measured after 30 days) was 3–4 orders greater than the pristine EP coating. The pristine EP coated sample displayed severe corrosion, whereas no evident corrosion product formation happened on the composite-coated sample [79]. In earlier work, the authors used a homogeneous dispersion of BN quantum dots (QDs) and BN nanoplates for depositing a waterborne EP composite coating. TEM images revealed that the h-BN nanoplatelets absorbed the QDs; the nanoplatelets aggregation in water was subdued in the presence of the QDs. QDs - BN addition in the matrix led to considerably distinct fracture morphology and suppressed crack propagation. The oxygen and nitrogen gas permeability diminished with an upsurge of the QDs - BN's amount in the EP. The improved corrosion resistance was credited to the better barrier effect of the insulating h-BN and the better compatibility and interface bonding with the EP matrix [78].

Most reports on EP composite coating used *spraying/painting* for coating fabrication, as discussed below. To make the BNNSs dispersed and effectively interacted with the EP matrix, various chemical functionalizations were attempted, including amine-capped aniline trimer (ACT) [80,81], polyethyleneimine (PEI) [82,83], PDA [84] and ionic liquids [85]. Composite coatings of EP with nanocarbons [[86], [87], [88]] and conducting polymers [[89], [90], [91], [92]] were also explored.

Cui et al. utilized π–π interactions of aromatic rings of *ACT* with BNNSs to make a stable dispersion of BNNSs in an organic solvent. The ACT incorporation into the EP resin through curing improved the EP - BNNSs interfacial interactions. Instead, ACT itself acted as a surface passivator. Electrochemical studies showed that 1.0 wt% of the BNNSs-added coating had the top corrosion protection in 3.5 wt% NaCl. A 0.5 wt% EP - BNNSs coating displayed the lowermost wear rate and depths. At a higher BNNSs content (2.0 wt%), more cracks and defects appeared on the fracture surface, which was attributed to the NS's aggregation [81]. In subsequent work, the authors showed that introducing well-dispersed functionalized BNNSs into a waterborne EP considerably improved corrosion protection. The composite-coated sample provided a long-term protective effect when the pristine EP coating swelled after 420 h immersion [80].

**Fig. 9:**
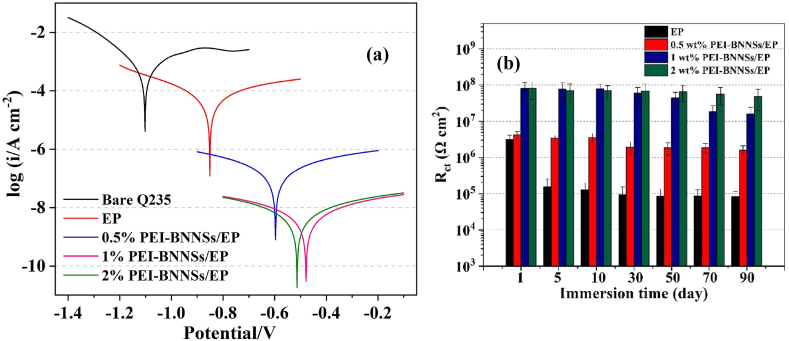
**(a)** Potentiodynamic polarization curves and **(b)***R*_ct_ values obtained from EIS plots after 50 days of 3.5% NaCl immersion. *Reproduced with permission from**Ref. [82]**© 2020 Elsevier B.V.*

*PEI modification* was shown to be critical in boosting the anticorrosion property of EP - BNNSs coating [[82], [83], [84]]. Lewis bases, such as amines, can complex with electron-deficient B atoms of BNNSs [82,93]. The amino group of PEI - BNNSs can enhance interfacial interactions within the EP matrix during curing. A composite coating of PEI - BNNSs - EP presented admirable long-term corrosion protection for P110 mild steel. The impedance at 0.01 Hz of the composite coated mild steel after 70 days of immersion in 3.5 wt% NaCl was 6.63 × 10^7^ Ω cm^2^ [83]. h-BN powder was ultrasonicated in an aqueous PEI solution to simultaneously exfoliate and functionalize, and used as nanofillers for waterborne EP coating for Q235 carbon steel. SEM images of fractured surfaces revealed nearly no PEI - BNNSs agglomeration. The *i*_corr_ determined from Tafel analysis of 1 and 2 wt% of PEI - BNNSs added EP coated samples were ∼4 orders of magnitude inferior to neat EP (Fig. 9a). The *R*_ct_ of neat EP reduced from 3.16 × 10^6^ to 8.16 × 10^4^ Ω cm^2^ after 90 days, whereas *R*_ct_ of the 2% PEI - BNNSs - EP remained at ∼4.81 × 10^7^ Ω cm^2^ (Fig. 9b) [82]. An in situ hydrothermal made PDA - BNNSs - CeO_2_ nanohybrid was used as a corrosion-inhibiting filler in EP resin, where CeO_2_ acted as the inhibitor. Localized red rust formation happened on the BNNS - EP coating after 15 days in 3.5 wt% NaCl, whereas no corrosion product formation or delamination happened on the surface of the PDA - BNNSs - CeO_2_ - EP coated sample [84]. CeO_2_ - BNNSs filler dispersed into aqueous EP provided excellent corrosion resistance to Q345 steel. After 20 days of immersion into 3.5 wt% NaCl solution, the composite coating can keep an impedance modulus of 9.61 × 10^7^ Ω cm^2^ at 0.01 Hz, which was larger than those of the neat EP and the EP - BNNSs coating [94].

**Fig. 10:**
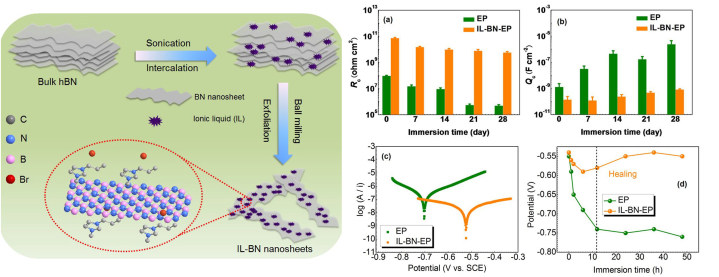
**(Left)** Schematic of exfoliation of h-BN via IL-assisted ball-milling. **(Right) (a, b)** Coating resistance and capacitance as a function of immersion. **(c)** Potentiodynamic polarization plots and **(d)** Corrosion potential variation. *Reproduced with permission from**Ref. [85]**© 2021 American Chemical Society.*

*Ionic liquids (ILs)* have attracted considerable research curiosity due to their excellent properties [95]. They are also known for their corrosion inhibition capability [96]. They can be good dispersing agents, as the conjugated structure and N atoms could ease π−π interactions with BNNSs. Du et al. synthesized ultrathin BNNSs and simultaneously noncovalently functionalized them by IL (Fig. 10). 5 mg/mL IL - BNNSs aqueous dispersion was mixed with 12 g waterborne EP (0.5 wt%) and 6 g curing agent, bar coated on Q235 steel and room temperature cured (coating thickness ∼28 μm). After salt water immersion for 4 weeks, the composite-coated sample presented a high coating resistance of 5.6 × 10^9^ Ω cm^2^, which was 4 orders greater than the neat EP-coated sample, owing to the combined effect of the improved physical barrier effect and the IL's passivation/self-healing effect (Fig. 10a–d). 300 h of salt-spray studies on scratched samples showed that neat EP-coated sample suffered from severe corrosion with coating delamination, whereas the IL - BNNSs - EP sample showed only minor deterioration [85].

A few works used *nanocarbons* in EP - BNNSs coating formulation [[86], [87], [88]]. Wang et al. grafted BNNSs on PDA-modified carbon fiber (CF) and used them as fillers. Compared to neat EP, the interfacial shear and tensile strengths of the BNNSs - CF - EP composite were augmented by 87.2% and 13.8%, respectively. The BNNSs - CF - EP composite displayed an 83.0% drop in wear rate and a 37.8% drop in friction coefficient. EIS studies after 40 days in 3.5 wt% NaCl showed that the modulus of impedance at 0.01 Hz of BNNSs - CF - EP, EP - CF, EP - BN, and plain EP coatings were 6.25 × 10^8^, 5.60 × 10^6^, 6.05 × 10^6^ and 4.15 × 10^6^ Ω cm^2^, respectively [88]. GO can work as an intercalator assisting h-BN exfoliation and boost the BNNSs dispersion in waterborne EP. The π-π interaction between GO and h-BN could allow homogeneous stacking of BNNSs on GO. The surface images of coated samples after 240 h salt spray exposure showed evident pits and bubbles formation around the artificial scratches on neat EP coating, whereas only fewer bubbles and corrosion products appeared on BNNSs - GO - EP composite coated sample [87]. Yu et al. showed that APTES-modified 0.5 wt% BNNSs - EP waterborne coating had a better anticorrosive ability for Q235 steel compared to the corresponding GR and GO counterparts [86].

**Fig. 11:**
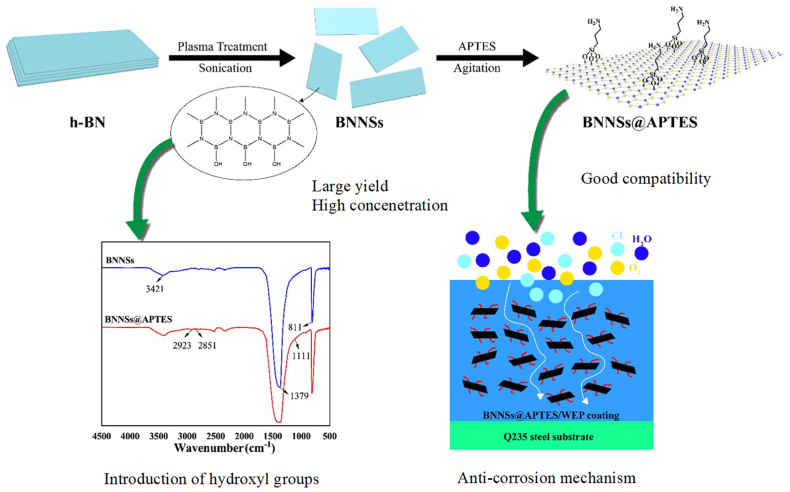
Fabrication schematic and FTIR spectra of BNNSs - APTES and a protection mechanism of the BNNSs - APTES - EP coating. *Reproduced with permission from**Ref. [99]**© 2022 Elsevier B.V.*

Several works used low surface energy compounds to enhance the hydrophobicity of the coated surface. Silane-functionalized EP and PDA-functionalized BNNSs were used in a formulation [97]. Li et al. investigated the role of polytetrafluoroethylene (PTFE) on the corrosion and tribological resistance of EP - hydroxylated h-BN coating (thickness ∼25 μm). Adding PTFE enhanced the hydrophobicity with a water contact angle of up to 114.3^°^. The average wear rate and friction coefficient of the EP - BN - PTFE coating (PTFE/EP mass ratio - 0.8) diminished by 60.3% and 68.4% compared to EP - BN coating. The EP - BN - PTFE coated sample displayed a high impedance modulus of 2.28 × 10^11^ Ω cm^2^ (at 0.01 Hz) after 80 days of NaCl immersion, which was ∼5 times that of EP - BN coating, emphasizing the importance of the PTFE incorporation [98]. h-BN was exfoliated into BNNSs by calcination, air plasma treatment, and sonication in DMF. BNNSs with attached hydroxyl groups were further modified by APTES (Fig. 11). The contact angles of neat EP and 0.8 wt% BNNSs - APTES - EP coating were ∼71.7^°^ and 90.4^°^, respectively. The BNNSs - APTES created the labyrinth effect in the matrix, making tortuous diffusion pathways for the corrosive medium and enhancing the barrier properties (Fig. 11) [99].

Tannic acid is known for its rust conversion properties [100,101]. CVD-made BNNSs were modified by tannic acid (T-BNNS) and incorporated in EP, which resulted in significantly improved mechanical tensile strength (15.10 MPa), thermal conductivity (0.45 W/m·K) and much-improved corrosion resistance. The *i*_corr_ of the 10% T-BNNS - EP coating in 3.5 wt% NaCl was 8.053 × 10^−7^ A/cm^2^. The impedance at 0.01 Hz of the composite coating was much greater than neat EP coating during 120 h of immersion [102]. Shi et al. decorated BNNSs with magnetic ZnFe_2_O_4_, and modified them with stearic acid to attain flame-retardant and hydrophobic characteristics. Under a magnetic field, the ZnFe_2_O_4_ - BNNSs can achieve a well-ordered alignment in the EP matrix. A 10% ZnFe_2_O_4_ - BNNSs - EP coating reduced the CO production and peak heat release rate by 51% and 47%, respectively, and provided surface superhydrophobicity. The coated surface reduced the friction coefficient by 76.9% [103]. BNNSs could act as a container to hold corrosion inhibitors to a certain extent facilitating a slow release [104].

A few works explored *conductive polymers with* EP - BNNS [[89], [90], [91], [92]]. The protection by conducting polymers is typically attributed to the combined effect of anodic protection, barrier protection, electrochemical switching and corrosion inhibition [105,106]. Polyaniline (PAni) - h-BN nanocomposites were made via in situ polymerization of aniline on BNNSs, and used in waterborne EP coating. PAni helped enhance NS's dispersion in the EP resin and concurrently acted as a corrosion inhibitor [91]. PAni - BN covalent linking was realized using *p*-aminobenzoic acid via in situ polymerization, mixed with a silane solution, sonicated, integrated into a water-based EP resin formulation and coated on hot-dip galvanized steel (coating thickness ∼5 μm). The initial impedance modulus of the PAni - BN - EP, PAni - EP, BN - EP and neat EP coated samples were 7.67 × 10^5^, 8.73 × 10^3^, 3.32 × 10^4^ and 2.94 × 10^3^ Ω cm^2^, respectively. The improved corrosion protection by the composite coating was ascribed to the decreased coating defects, enhanced barrier effect, added oxide formation on galvanized layer by PANI, and ATMP ion's release during de-doping [90]. BNNSs were obtained by h-BN exfoliation with poly(2-butyl aniline), amalgamated into EP coating, bar coated on Q235 steel and cured at 80 °C (thickness ∼20 μm). The surface passivation by the electroactive poly(2-butyl aniline) also contributed to enhanced protection [89]. Lu et al. functionalized BNNSs with polypyrrole and incorporated them into EP. A 0.5 wt% modified-BNNSs incorporated EP coating presented ∼58 times lower corrosion rate than neat EP during 40 days of 3.5 wt% NaCl immersion [92].

#### 3.3.2 Polyurethane coatings

Polyurethanes (PUs) have been extensively used in adhesives, leathers, textiles and protective coatings. Several works incorporated BNNSs into PU coatings to enhance performance [[107], [108], [109]]. Li et al. functionalized hydroxylated BN with *p*-phenylenediamine modified reduced-GO and used them as fillers in PU coating. The tensile modulus and strength of the composite film showed 95% and 62% improvement, compared to neat PU film, attributed to the effective interfacial interactions. The impedance modulus at 0.01 Hz of neat and the composite coated samples after 7 days of NaCl immersion were 4.27 × 10^4^ and 1.74 × 10^7^ Ω cm^2^, respectively [108].

**Fig. 12:**
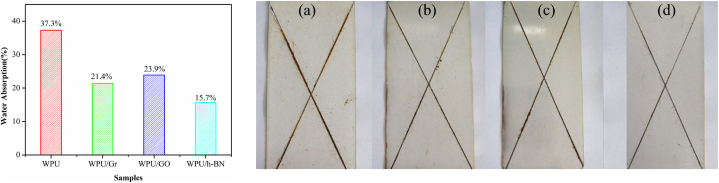
(a) Water absorption results and **(b)** Photographs of crosscut samples after 72 h of 0.5 mol/L NaCl immersion: (a) neat PU, (b) PU - GR, (c) PU - GO and (d) PU–BN composite coatings. *Reproduced with permission from**Ref. [109]**© 2020 Taylor & Francis Group, LLC.*

A comparative study on the reinforcement (in-situ polymerization, loading amount - 0.25 wt%) of h-BN, GR and GO to waterborne PU showed that the rate of water absorption of BN – PU reduced from 37.3 to 15.7%, and the tensile strength amended from 4.16 to 19.56 MPa. h-BN was the best nanofiller for PU, better than GR and GO (Fig. 12a). After 72 h of 0.5 mol/L NaCl immersion, several corrosion pits were visible along the crosscut of the neat PU coated sample compared to the composite coated sample (Fig. 12a–d). The poor dispersion and galvanic corrosion were responsible for GR and GO's inferior performance as discussed in previous sections [109]. Waterborne PU dispersion was prepared using a trimethoxysilane end-capping agent derived from diallylamine, (3 glycidoxypropyl) methyldiethoxysilane and modified with edge-hydroxylated BNNSs (0.2 wt%). The water contact angle of the composite coated sample was 101.2^°^; the water absorption was reduced by 52% compared to neat PU [107]. Table S1 lists BNNSs-incorporated composite protective coatings and their corrosion protection performance.

#### 3.3.3 Other polymer coatings

BNNSs were also shown to be an adequate filler with other polymer coatings. Reported works include BNNSs - Acrylic for 304 SS [110], KH560-modified BNNSs - Acrylic on galvanized steel [111], plasma treated BNNSs - Acrylic for low carbon steel [112], phenyl silane functionalized BNNSs - Poly(arylene ether nitrile) for P110 steel [113], BNNSs - Polyvinyl alcohol (PVA) for 316L SS [114], BNNSs - Poly vinyl butylene for Cu [115], PDA-functionalized BNNSs - PVA for 304 SS [116], PDA - GO - BNNSs - Polyvinylbutyral for mild steel [117], TiO_2_ decorated BNNSs - Poly (3,4-ethylene dioxythiophene) for 316 LSS [118], BNNSs-incorporated polyisobutylene [119], and BNNSs - Cellulose acetate [120] and BNNSs - PVA [121] for preventing polymer degradation.

**Fig. 13:**
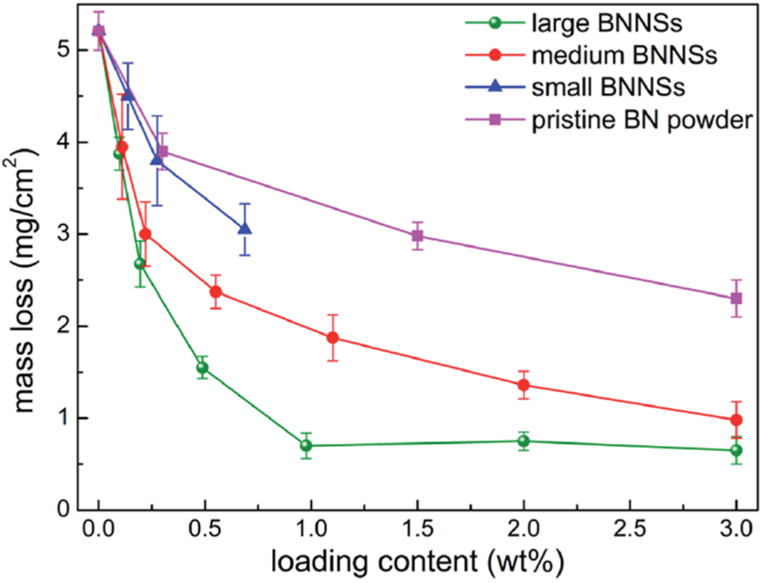
Mass loss due to oxygen-atom corrosion of PVA - BNNSs films incorporated with different lateral-sized BNNSs. *Reproduced with permission from**Ref. [121]**© 2015 The Royal Society of Chemistry.*

Sun et al. showed that the corrosion protection of a poly(vinyl butylene) - BNNSs coating (1.0 wt%) was ∼67000 times higher than that of pristine poly(vinyl butylene) coating [115]. The significant enhancement of corrosion protection of a plasma-treated BNNSs - Acrylic coating was ascribed to the enlarged interlayer spacing of h-BN and formation of more exfoliated/unparalleled h-BN nanoflakes by the plasma treatment, blockage of pin-holes by the better-dispersed BNNSs, and the existence of more –OH/–NH surface groups, which can capture more Fe^2+^ ions [112]. A study by Yi et al. has shown that the efficiency of BNNSs to protect polymer's oxygen-atom corrosion varies with their average lateral size (Fig. 13). Here, BNNSs - PVA dispersions with different lateral-sized BNNSs (thickness < 3 nm) were used. The enhancement was attributed to the much fewer edges of larger BNNSs and less penetrative channels than smaller ones (better barrier effect) and the efficient oxygen-healing mechanism of BNNSs with N vacancies (bonding effect) [121].

### 3.4 Others

BNNSs were utilized in various electrochemical energy storage systems [122]. An h-BN-based composite coating effectively improved Ni-rich cathodes' cycle stability and safety for high energy lithium-ion batteries [123]. BNNSs were effective as a binder-free oxidation and fire-resistant wood coating [124] and enhanced the fireproof performance of construction coatings [125]. A few recent works addressed h-BN-based thermal spray coatings [[126], [127], [128]].

Several works focused on anti-biofouling and anti-bacterial coatings. The cellular interaction of the 2D materials-incorporated layers with biological matter and the influence of their crystallinity and surface roughness need further addressing in deriving biologically relevant biocompatible and anti-biofouling coatings in different applications. This area has not been discussed in this review. More details of BNNS's interaction with living matter can be found elsewhere [[129], [130], [131], [132], [133], [134], [135]].

## 4 Conclusions and perspectives

This review accounts for the state-of-art research on 2D h-BN in anti-corrosion, anti-oxidation and anti-wear protective coatings. The 2D nanomaterials have several outstanding properties, as discussed in this review. BN is advantageous in protective coatings primarily due to its good mechanical properties, high thermal stability and electrical insulating characteristics. Vapour-deposited h-BN thin films and exfoliated BNNSs-incorporated composite coatings are detailed.

Section 2 focuses on vapour-deposited h-BN thin films. Most of the works in this category explored monolayer, few-layer or multilayer CVD coatings for anti-corrosion and anti-oxidation applications, particularly for Cu. Most studies showed that the CVD monolayer h-BN is ineffective for oxidation or corrosion protection in the longer term. This can be associated with the difficulty in achieving 100% surface coverage with a monolayer and the greater susceptibility to oxidation via grain boundaries and defects. On the other hand, a multilayer coating could cover the defects more effectively, and the oxidation happens slowly, mainly via the wrinkle's region. A few studies showed that few-layer coatings are better than their thicker counterparts as the defect density associated with the latter was more significant. Precise optimization of the layer thickness is essential. On the other hand, monolayer h-BN is shown to be effective for anti-bacterial applications. They can resist biocorrosion to Cu effectively. More studies can be attempted for CVD h-BN coatings for different applications of stainless steel and other alloys. A few recent reports on PVD coatings are available. Most studies showed that the protection performance of h-BN is better than the corresponding GR due to the former's better thermal stability and insulating characteristics. A few works used exfoliated BNNSs to fabricate BN coatings.

Section 3 discusses BNNSs-incorporated inorganic, nanocarbon and polymer nanocomposite coatings. BNNSs are efficient fillers for protective coatings as the sheet-form structures can cover the holes and cracks and postpone surface degradation. The robustness and the insulating characteristics are advantageous over GR. The hybrid coatings of BNNSs, along with nanocarbons, are excellent anti-wear coatings. GR – BNNSs - MoS_2_ hybrid can be an attractive candidate where GR and MoS_2_ can provide anti-wear properties and BNNSs provide the barrier effect. More than 80% of reports with BNNSs - Polymer nanocomposite coating used epoxy resin, followed by polyurethanes and others for anti-corrosion applications. Various chemical functionalizations were attempted, including polyethyleneimine, poly(dopamine) and ionic liquids. Most studies showed that incorporating 0.5 to 2.0 wt% BNNSs in the formulation yielded better results, above which severe agglomeration happens. A third-party component such as ionic liquids, PTFE and tannic acid in the BNNSs - EP coating significantly enhanced the protection properties. They can be used in the exfoliation process to achieve simultaneous exfoliation and functionalization. A few works showed that BNNS film protect polymers from oxygen-atom corrosion.

The 2D-materials incorporated coatings are expected to have complex interactions with the coating matrix depending on the morphological and electronic properties and surface features such as wettability. As discussed above, BNNS outperform GR majorly due to the insulating characteristics. Several studies are available in this direction. A few studies compared BNNS to MoS_2_. More studies comparing different 2D materials, including MXenes in protective coatings, could be fruitful.

The stability and performance of BNNSs ultimately depend on the quality of the NSs, demanding precise optimization in the preparation methods and formulation of standards and protocols. Cheaper and greener industrial-scale exfoliation and direct surface modification methods could be designed. More effective approaches favouring their strong substrate adhesion and effective cross-linking within the coating matrix could be beneficial.

## Author contribution statement

All authors listed have significantly contributed to the development and the writing of this article.

## Data availability statement

No data was used for the research described in the article.

## Declaration of competing interest

The authors declare that they have no known competing financial interests or personal relationships that could have appeared to influence the work reported in this paper.
