# Supplementary material for: 2D hexagonal boron nitride (h-BN) nanosheets in protective coatings: A literature review
Source: Heliyon. 2023 Aug 25;9(9):e19362. doi: 10.1016/j.heliyon.2023.e19362 (PMC10481311; doi:10.1016/j.heliyon.2023.e19362)
Supplement: Multimedia component 1 [file mmc1.docx]

**Table S1:** A list of reported works on BNNSs-based composite anti-corrosion coatings.

| **Coating composition** | **Coating type** | **Substrate** | **Electrochemical studies** | | **Ref.** |
| --- | --- | --- | --- | --- | --- |
|  |  |  | **Electrolyte** | **Corrosion parameters** |  |
| BNNSs - Zinc phosphate | Chemical conversion | Q235 carbon steel | 3.5 wt% NaCl | *I*_corr_ = 3.21 × 10^−6^ A/cm^2^  CR = 0.47 mpy  *R*_ct_  = 4.88 × 10^4^ Ω.cm^2^  *R*_c_  = 2.84 × 10^3^ Ω.cm^2^ | [58] |
| PDA modified BNNSs - Zinc phosphate - Silane | Chemical conversion | Q235 carbon steel | 3.5 wt% NaCl | *I*_corr_ = 1.95 × 10^−5^ A/cm^2^  CR = 8.93 mpy  *R*_ct_  = 1183 Ω.cm^2^  *R*_c_  = 1676 Ω.cm^2^ | [59] |
| BNNSs - MoS_2_ | Chemical conversion | Mild steel | 3.5 wt% NaCl | *I*_corr_ = 13.84 ×10^−6^ A/cm^2^  CR = 0.161 mm/year  *R*_ct_  = 1596 Ω  *R*_c_  = 1159 Ω | [61] |
| BNNSs - Nickel | Pulse ED | 304 SS | 3.5 wt% NaCl | *I*_corr_ = 8.76 × 10^−7^ A/cm^2^  CR = 0.0094 mm/year | [64] |
| BNNSs - Chitosan | EPD | PEO / Mg alloy | SBF | *I*_corr_ = 7.56 × 10^–6^ A/cm^2^  *R*_ct_  = 717.10 Ω.cm^2^  *R*_c_  = 2432 Ω.cm^2^ | [65] |
| Phosphorylated BNNSs | EPD | Q235 carbon steel | 3.5 wt% NaCl | *I*_corr_ = 0.549 × 10^–6^ A/cm^2^  *R*_ct_  = 101.2 × 10^3^ Ω.cm^2^  *R*_c_ = 10.1 × 10^3^ Ω.cm^2^ | [66] |
| BNNSs - Zirconium oxide | Sol-gel | 316 L SS | 3.5 wt% NaCl | *R*_ct_  ~ 10^6^ Ω.cm^2^  *R*_c_ ~ 10^6^ Ω.cm^2^ | [67] |
| BNNSs-GR | Spray coating | Mild steel | 3.5 wt% NaCl | *I*_corr_ = 3.37 × 10^–7^ A/cm^2^  CR = 3.97 × 10^-3^ mm/year | [71] |
| *Polymer coatings (EP and PU-based)* | | | | | |
| BN quantum dots - BNNSs - EP | Blade coating | Q235 carbon steel | 3.5 wt% NaCl | *I*_corr_ = 5.57 × 10^–9^ A/cm^2^  CR = 6.48 × 10^-5^ mm/year  *Z*_0.01 Hz_ ~ 10^8^ Ω.cm^2^ | [78] |
| Amino-functionalized BNNSs - EP | EPD | Q235 carbon steel | 3.5 wt% NaCl | *Z*_0.01 Hz_ *=* 5.83 × 10^10^ Ω.cm^2^  *R*_ct_  ~ 10^10^ Ω.cm^2^  *R*_ct_ ~ 10^10^ Ω.cm^2^  (after 30 days of immersion) | [79] |
| ACT functionalized BNNSs - EP | Bar coating | Q235 carbon steel | 3.5 wt% NaCl | *Z*_0.01 Hz_ ~ 10^10^ Ω.cm^2^  *R*_ct_ ~ 10^10^ Ω.cm^2^  (after 60 days of immersion) | [81] |
| PEI modified BNNSs - EP | Bar coating | Q235 carbon steel | 3.5 wt% NaCl | *I*_corr_ = 3.41 × 10^–9^ A/cm^2^  *Z*_0.01 Hz_ *=* 6.96 × 10^7^ Ω.cm^2^ *R*_ct_ ~ 10^8^ Ω·cm^2^  *R*_c_ ~ 10^7^ Ω·cm^2^  (after 90 days of immersion) | [82] |
| PEI modified BNNSs - EP | Spraying | P110 carbon steel | 3.5 wt% NaCl | *I*_corr_ = 2.99 × 10^–10^ A/cm^2^  *Z*_0.01 Hz_ *=* 2.45 x 10^9^ Ω.cm^2^ | [83] |
| PDA modified BNNSs - CeO_2_ - EP | Bar coating | Carbon steel | 3.5 wt% NaCl | *Z*_0.01 Hz_ *=* ~ 10^9^ Ω.cm^2^  *R*_ct_ = 3.37 x 10^7^ Ω·cm^2^  *R*_c_ = 7.49 x 10^8^ Ω·cm^2^  (after 35 days of immersion | [84] |
| IL - BNNSs - EP | Bar coating | Q235 carbon steel | 3.5 wt% NaCl | *I*_corr_ = 7.1 × 10^–8^ A/cm^2^  CR = 8.3 × 10^-4^ mm/year  *Z*_0.01 Hz_ *=* 3.7 x 10^10^ Ω.cm^2^  *R*_c_ ~ 10^10^ Ω·cm^2^ | [85] |
| BNNSs - GO - EP | Spray coating | P110 carbon steel | 3.5 wt% NaCl | *R*_c_ = 4.05 x 10^6^ Ω·cm^2^  (after 2 h of immersion)  *R*_ct_ = 1.75 x 10^5^ Ω·cm^2^  (after 10 days of immersion) | [87] |
| APTES modified BNNS - EP | Bar coating | Q235 carbon steel | 3.5 wt% NaCl | *I*_corr_ = 4.96 × 10^–8^ A/cm^2^  *Z*_0.01 Hz_ *=* 1.48 x 10^7^ Ω.cm^2^  (after 1 day of immersion)  *Z*_0.01 Hz_ *=* 1.21 x 10^6^ Ω.cm^2^  (after 21 days of immersion) | [86] |
| BNNSs - PDA modified carbon fiber - EP | Bar coating | 316 L SS | 3.5 wt% NaCl | *Z*_0.01 Hz_ ~ 10^9^ Ω.cm^2^  (after 5 days of immersion)  *R*_ct_ ~ 10^8^ Ω·cm^2^  *R*_c_ ~ 10^8^ Ω·cm^2^  (after 40 days of immersion) | [88] |
| Hydroxylated BNNSs - PTFE - EP | Spray coating | Q235 carbon steel | 3.5 wt% NaCl | *Z*_0.01 Hz_ *~* 10^11^ Ω.cm^2^  (after 60 days of immersion)  *R*_ct_ ~ 10^12^ Ω·cm^2^  (after 60 days of immersion) | [98] |
| APTES modified BNNSs - EP | Spin coating | Q235 carbon steel | 3.5 wt% NaCl | *Z*_0.01 Hz_ *=* 8.50 x 10^9^ Ω.cm^2^  (after 10 h of immersion)  *Z*_0.01 Hz_ *=* 6.37. x 10^7^ Ω.cm^2^  (after 168 h of immersion)  *R*_ct_ ~ 10^8^ Ω·cm^2^  (after 168 h of immersion) | [99] |
| Tannic acid modified BNNSs - EP | Knife coating | Cold-rolled 304 SS | 3.5 wt% NaCl | *I*_corr_ = 8.053 × 10^−7^ A/cm^2^  *R*_c_ = 5.21 × 10^4^ Ω·cm^2^  (after 120 h of immersion) | [102] |
| BNNSs - zinc phosphate - EP | Bar coating | Mild steel | 3.5 wt% NaCl | *Z*_0.01 Hz_ *~* 10^7^ Ω.cm^2^  *R*_c_ ~ 10^7^ Ω·cm^2^  (after 15 days of immersion) | [104] |
| BNNSs - Polyaniline - EP | Solution casting | Tin | 3.5 wt% NaCl | *R*_c_ = 3.71 x 10^9^ Ω·cm^2^  (after 5 days of immersion)  *R*_c_ = 8.22 x 10^8^ Ω·cm^2^  (after 28 days of immersion) | [91] |
| BNNSs - Polyaniline - - EP | Bar coating | Hot-dip galvanized steel | 3.5 wt% NaCl | *R*_c_ = 6.01 x 10^5^ Ω·cm^2^  (after 1 h of immersion)  *R*_ct_ = 1.94 x 10^4^ Ω·cm^2^  (after 24 h of immersion | [90] |
| BNNSs - Poly(2-butyl aniline) - EP | Bar coating | Q235 carbon steel | 3.5 wt% NaCl | *Z*_0.01 Hz_ *=* 15.0 x 10^8^ Ω.cm^2^  (after 15 days of immersion)  *R*_c_ ~ 10^9^ Ω·cm^2^  (after 120 days of immersion) | [89] |
| BNNSs - Polypyrrole - EP | Spin coating | Q235 carbon steel | 3.5 wt% NaCl | *I*_corr_ = 1.3 × 10^−9^ A/cm^2^  *R*_c_ ~ 10^7^ Ω·cm^2^  (after 40 days of immersion)  *Z*_0.01 Hz_ *~* 10^7^ Ω.cm^2^ | [92] |
| Hydroxylated BNNSs - Phenylenediamine-modified reduced GO - PU | Solution casting | Steel | 3.5 wt% NaCl | *I*_corr_ = 5.26 × 10^−9^ A/cm^2^  CR = 6.14 × 10^−5^ mm/year  *Z*_0.01 Hz_ *=* 1.47 x 10^7^ Ω.cm^2^  (after 15 days of immersion) | [108] |
| BNNSs - PU | Bar coating | Galvanized steel | 5 g/L NaCl + 3.5 g/L (NH_4_)_2_SO_4_ | *I*_corr_ = 0.12 × 10^−6^ A/cm^2^ | [109] |
| Hydroxylated BNNSs – Trimethoxysilane - PU | Solution casting | Aluminum | 3.5 wt% NaCl | *I*_corr_ = 3.7 × 10^−10^ A/cm^2^  *Z*_0.01 Hz_ *=* ~ 10^6^ Ω | [107] |

***I_corr_*** *– Corrosion current density and* ***CR*** *– Corrosion rate from potentiodynamic polarisation plots.*

***R_ct_*** *&* ***R_c_*** – *Charge transfer resistance and Coating resistance from impedance spectroscopy studies.*

***Z_0.01 Hz_*** – *Impedance at 0.01 Hz*

***SS*** *– Stainless steel;* ***ED*** *– Electrodeposition;* ***EPD*** *– Electrophoretic deposition;* ***PEO*** *– Plasma electrolytic oxidation;*

***EP*** *– Epoxy;* ***APTES*** *– (3-Aminopropyl)triethoxysilane;* ***ACT*** *– Amine-capped aniline trimer;* ***PEI*** *– Polyethyleneimine;* ***PDA*** *– Polydopamine;* ***IL*** *– Ionic liquid.*
